# Neuroprotective Effects of Zinc Sulfate Against Type 2 Diabetes-Induced Encephalopathy in Aged Rats

**DOI:** 10.3390/ijms27167412

**Published:** 2026-08-19

**Authors:** Omer Unal, Nilufer Akgun-Unal, Emre Soner Tiryaki, Cemile Avci-Akan, Elif Gulbahce-Mutlu, Adem Atacak, Abdulkerim Kasim Baltaci

**Affiliations:** 1 Department of Physiology, Faculty of Medicine, Samsun University, Samsun 55080, Türkiye; 2 Department of Biophysics, Faculty of Medicine, Ondokuz Mayis University, Samsun 55270, Türkiye; nilufer.akgununal@omu.edu.tr; 3 Department of Physiology, Faculty of Medicine, Ondokuz Mayis University, Samsun 55139, Türkiye; emre.tiryaki@omu.edu.tr; 4 Department of Transport Services, Alaçam Vocational School of Higher Education, Ondokuz Mayıs University, Samsun 55800, Türkiye; cemile_avc@hotmail.com; 5 Department of Medical Biology, Faculty of Medicine, KTO Karatay University, Konya 42020, Türkiye; elif.mutlu@karatay.edu.tr; 6 Department of Physiology, Faculty of Medicine, Selcuk University, Konya 42130, Türkiye; adematacak04@gmail.com (A.A.); baltaci61@yahoo.com (A.K.B.)

**Keywords:** aging, T2DM, zinc sulfate, SIRT-1, BDNF, cerebral cortex, hippocampus

## Abstract

This study investigates the neuroprotective effects of zinc sulfate (ZnSO_4_) against diabetic encephalopathy in an aged female rat model of Type 2 diabetes mellitus (T2DM). T2DM was induced using a 4-week high-fat diet followed by a single 25 mg/kg STZ injection. Diabetic rats were treated with 10 mg/kg/day ZnSO_4_ for 4 weeks. We evaluated serum lipids, hippocampal oxidative stress (MDA, GSH), gene expressions (SIRT-1, GLUT3, BDNF, Bax, Bcl-2), and structural injuries (Nissl, PAS, GAP43, NGF) in the hippocampus and cerebral cortex. Results demonstrated that ZnSO_4_ significantly ameliorated systemic dyslipidemia. In the diabetic hippocampus, ZnSO_4_ mitigated oxidative stress by decreasing MDA and elevating depleted GSH levels. Molecularly, ZnSO_4_ upregulated the suppressed expressions of SIRT-1, GLUT3, BDNF, and Bcl-2, while downregulating pro-apoptotic Bax. Histopathological and immunohistochemical findings confirmed that ZnSO_4_ reduced neuronal degeneration and vascular pathologies (PAS positivity), preserving neuroplasticity (elevated GAP43 and NGF) in both brain regions. In conclusion, ZnSO_4_ supplementation provides potent, multifaceted neuroprotection against T2DM-associated neurodegeneration by regulating systemic dyslipidemia, restoring redox homeostasis, activating the SIRT-1/BDNF anti-apoptotic pathway, and preserving structural integrity.

## 1. Introduction

T2DM is a progressive metabolic disease marked by insulin resistance and disturbed glucose metabolism, which is widespread globally [1,2]. Research in the literature is increasingly focusing on the central nervous system (CNS) effects of long-term hyperglycemia and the ensuing cognitive decline, in addition to its well-documented cardiovascular, renal, and peripheral nervous system implications [3,4,5]. The prevalence of T2DM and the risk of diabetes-related cognitive decline spike significantly with age, especially in older age groups. Advanced age and chronic diabetic conditions combine to accelerate neurodegeneration, creating a foundation for the deterioration of the brain’s learning and memory functions, ultimately leading to diabetic encephalopathy [6,7]. Therefore, in this study, we define diabetic encephalopathy as the progressive deterioration of cognitive functions resulting from accelerated neurodegeneration, which is primarily driven by the synergistic destructive effects of advanced age, oxidative stress, neuroinflammation, and diabetes-induced zinc deficiency.

Age-related cognitive impairment and neurodegeneration associated with diabetes are primarily driven by oxidative stress and neuroinflammation, according to research [8]. Aging-related oxidative stress, combined with chronic hyperglycemia, results in an excessive production of reactive oxygen species (ROS) in brain tissue, thereby disrupting cellular redox balance [9]. The situation triggers a rise in lipid peroxidation, causing a substantial increase in malondialdehyde (MDA) levels and disrupting crucial cellular antioxidant processes, including reduced glutathione (GSH) [10]. The heightened oxidative stress burden sets off a toxic neuroinflammatory cascade that activates pro-inflammatory factors like NF-κB, thereby worsening age-related cellular damage in the hippocampus [2,8,11,12].

The Sirtuin 1 (SIRT-1) enzyme, a NAD^+^-dependent histone deacetylase that regulates cellular stress response, DNA repair, and senescence, plays a critical role in suppressing this neurotoxic process [13]. Under normal conditions, SIRT-1 activates antioxidant genes and strongly inhibits the inflammatory NF-κB signaling pathway. The stress resulting from aging and high blood glucose levels significantly downregulates SIRT-1 expression in hippocampal tissue, leaving neuronal cells highly vulnerable to neurodegeneration and inflammatory damage [13,14]. Insulin resistance additionally impairs the energy equilibrium of neurons by reducing the expression of glucose transporter 3 (GLUT3), thereby substantially lowering the production of brain-derived neurotrophic factor (BDNF), which is crucial for synaptic plasticity, learning capacity, and survival [15]. Disruption of the Bax/Bcl-2 balance in neurons by oxidative stress, inflammation, and reduced BDNF triggers the activation of caspase-3, thereby initiating irreversible apoptotic processes [16].

ZnSO_4_ supplementation is a notable potential treatment for diabetic neurodegeneration due to its action on multiple cellular targets. Zinc is an essential trace element found in high concentrations in the brain, particularly in cognitive areas such as the hippocampus and amygdala, and is crucial for neurogenesis, synaptic transmission, and cell survival [17]. The literature suggests that a considerable zinc deficiency (hypozincemia) occurs in diabetic patients because of hyperglycemia and increased zinc excretion in urine; this deficiency also worsens oxidative stress, learning difficulties, and psychiatric conditions such as depression and anxiety [18]. Research has shown that zinc supplementation, a highly effective antioxidant that does not readily participate in redox reactions, suppresses NADPH oxidase activity, boosts levels of endogenous antioxidants like MT (metallothionein) and GSH, and decreases the levels of malondialdehyde (MDA), a major marker of lipid peroxidation [10]. ZnSO_4_ administration has been reported to support the SIRT-1/BDNF pathway in diabetic animal models, thereby preserving synaptic plasticity, inhibiting apoptosis, and potentially reversing neurocognitive/behavioral impairment (depression and anxiety) [7,10,19]. The primary objective of this study is to explore the potential neuroprotective effects of ZnSO_4_ treatment against age-related neurodegeneration in a T2DM model. Specifically, we aimed to investigate the modulatory effects of ZnSO_4_ on oxidative stress, cellular energy metabolism, synaptic plasticity, and apoptosis in the brain. Furthermore, we sought to elucidate the therapeutic mechanisms of zinc against diabetic encephalopathy by evaluating its ability to preserve structural and cellular integrity in hippocampal and cortical tissues.

## 2. Results

### 2.1. Effects of ZnSO_4_ on Systemic Lipid Profile

The serum lipid profiles of the DM group showed statistically significant increases (*p* < 0.0001) in total cholesterol, triglycerides, and LDL levels compared to the CON group, with values of 95 ± 8, 110 ± 10, and 30 ± 4 mg/dL, respectively. In contrast, the HDL level in the DM group (12 ± 2 mg/dL) was significantly lower (*p* = 0.0001) than that in the CON group (35 ± 4 mg/dL). Compared to the DM group, the DM + ZnSO_4_ group treated with ZnSO_4_ showed significant decreases (*p* < 0.0001) in total cholesterol (120 ± 12 mg/dL), triglycerides (160 ± 15 mg/dL), and LDL (50 ± 6 mg/dL) levels, and significant increases (*p* = 0.0001) in HDL levels (25 ± 3 mg/dL) (Table 1).

### 2.2. Effects of ZnSO_4_ on Hippocampal Oxidative Stress Markers

**Figure 1:**
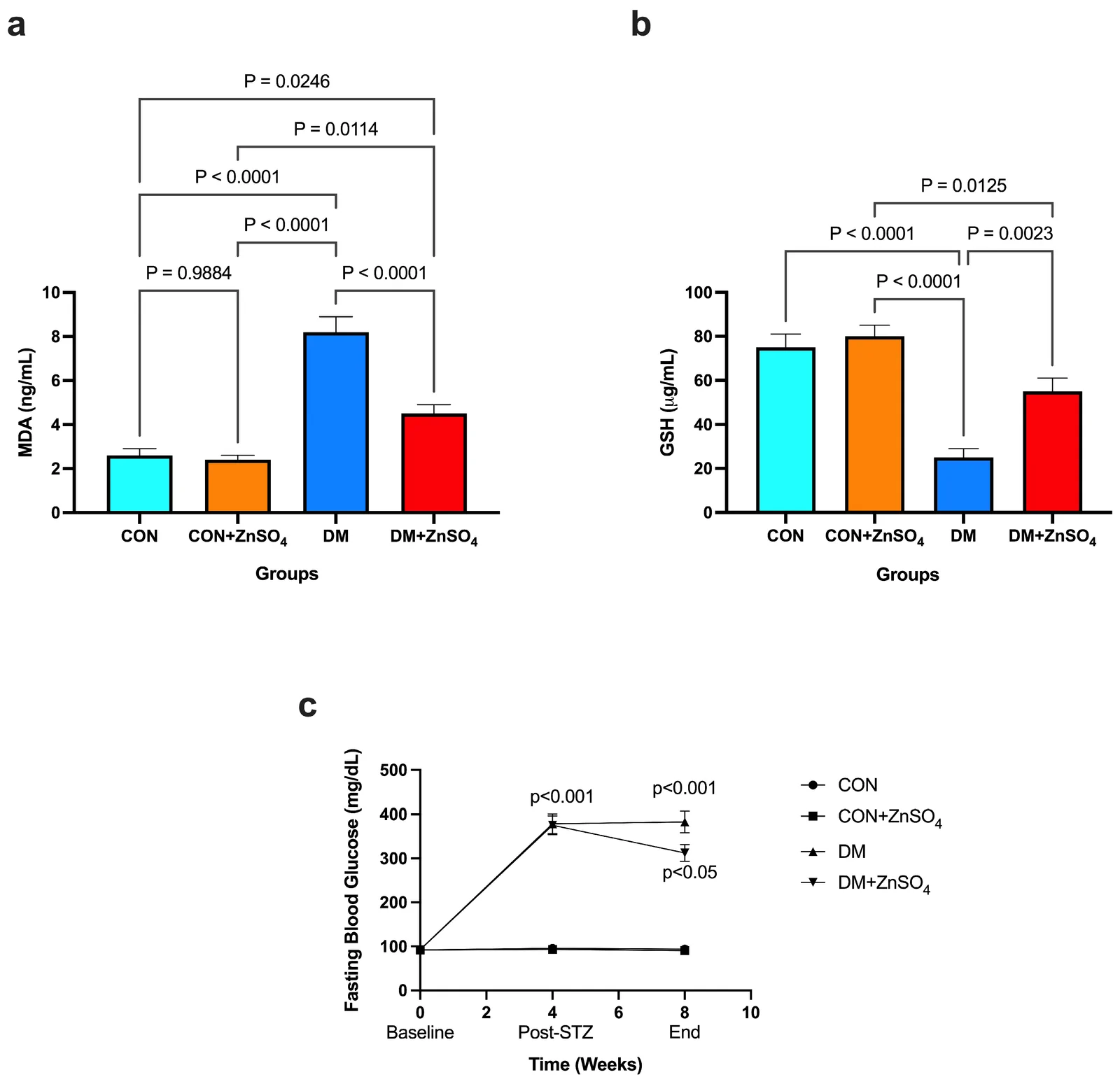
Effects of *ZnSO*_4_ treatment on hippocampal oxidative stress markers and chronological glycemia in an aged T2DM rat model. (**a**) Tissue malondialdehyde (MDA) levels (nmol/mg protein), a marker of cell membrane lipid peroxidation. (**b**) Reduced glutathione (GSH) levels (µg/mg protein), a key component of the endogenous antioxidant system. (**c**) Fasting blood glucose (glycemia) levels (mg/dL) monitored chronologically at baseline (Week 0), post-induction (Week 4), and at the end of the experiment (Week 8). Individual experimental groups are represented by distinct, high-contrast color-coded lines and bars (CON: turquoise, CON+*ZnSO*_4_: orange, DM: blue, and DM+*ZnSO*_4_: red) to ensure maximum visual distinction. The exact *p*-values indicating statistically significant differences between groups are shown directly on the respective figure panels. The exact *p*-values indicating statistically significant differences between groups are shown directly on the respective figure panels. Data are expressed as Mean ± Standard Error of the Mean (SEM) (*n* = 8).

When examining MDA levels—the end product of lipid peroxidation measured in hippocampal tissue homogenates—it was found that MDA levels in the DM group were statistically significantly higher (*p* < 0.0001) than those in the CON group. In the DM + ZnSO_4_ group, which received ZnSO4 treatment, MDA levels were found to be significantly lower (*p* < 0.0001) than those in the DM group. MDA levels in the CON + ZnSO_4_ group, which received only ZnSO_4_, were similar to those in the CON group (Figure 1a).

Significantly lower (*p* < 0.0001) reduced glutathione (GSH) levels, a crucial component of the cellular antioxidant defense system, were observed in the hippocampus of the DM group compared to the CON group. In contrast, the DM + ZnSO_4_ group, which received ZnSO4 supplementation, showed significantly higher (*p* < 0.0001) GSH levels compared to the untreated DM group. GSH levels in the CON + ZnSO_4_ group did not differ significantly from those in the CON group (Figure 1b).

Chronological fasting blood glucose (glycemia) levels were monitored at three critical phases of the experimental timeline: baseline (beginning of the experiment, Week 0), post-induction (Week 4, 72 h post-STZ injection), and at the end of the experiment (Week 8, at sacrifice). At baseline (Week 0), all experimental rats exhibited entirely physiological, normoglycemic levels with no statistically significant differences among the groups (CON: 92.4 ± 4.8 mg/dL; CON+ZnSO_4_: 91.8 ± 4.2 mg/dL; DM: 93.1 ± 5.1 mg/dL; DM+ZnSO_4_: 92.7 ± 4.6 mg/dL). Following 4 weeks of HFD feeding and a single low-dose STZ injection (Week 4), both the DM group (378.4 ± 22.6 mg/dL) and the DM+ZnSO_4_ group (375.2 ± 21.3 mg/dL) showed a highly significant and marked increase in fasting blood glucose compared to the healthy CON group (95.6 ± 5.2 mg/dL, *p* < 0.001), confirming successful diabetic induction. At the end of the experiment (Week 8, after 4 weeks of daily treatment), the fasting blood glucose in the untreated DM group remained severely elevated at 382.5 ± 24.8 mg/dL. In contrast, 4 weeks of daily ZnSO_4_ administration (10 mg/kg/day, i.p.) led to a statistically significant, moderate reduction in glycemia in the DM+ZnSO_4_ group (312.4 ± 19.5 mg/dL) compared to the untreated DM group (*p* < 0.05), indicating a partial insulin-sensitizing and glucose-lowering adjuvant benefit of zinc. The healthy control groups (CON and CON+ZnSO_4_) maintained stable, normoglycemic levels throughout the entire 8-week study period (CON: 94.1 ± 4.5 mg/dL; CON+ZnSO_4_: 90.8 ± 4.1 mg/dL) (Figure 1c).

### 2.3. Molecular and Gene Expression Findings

**Figure 2:**
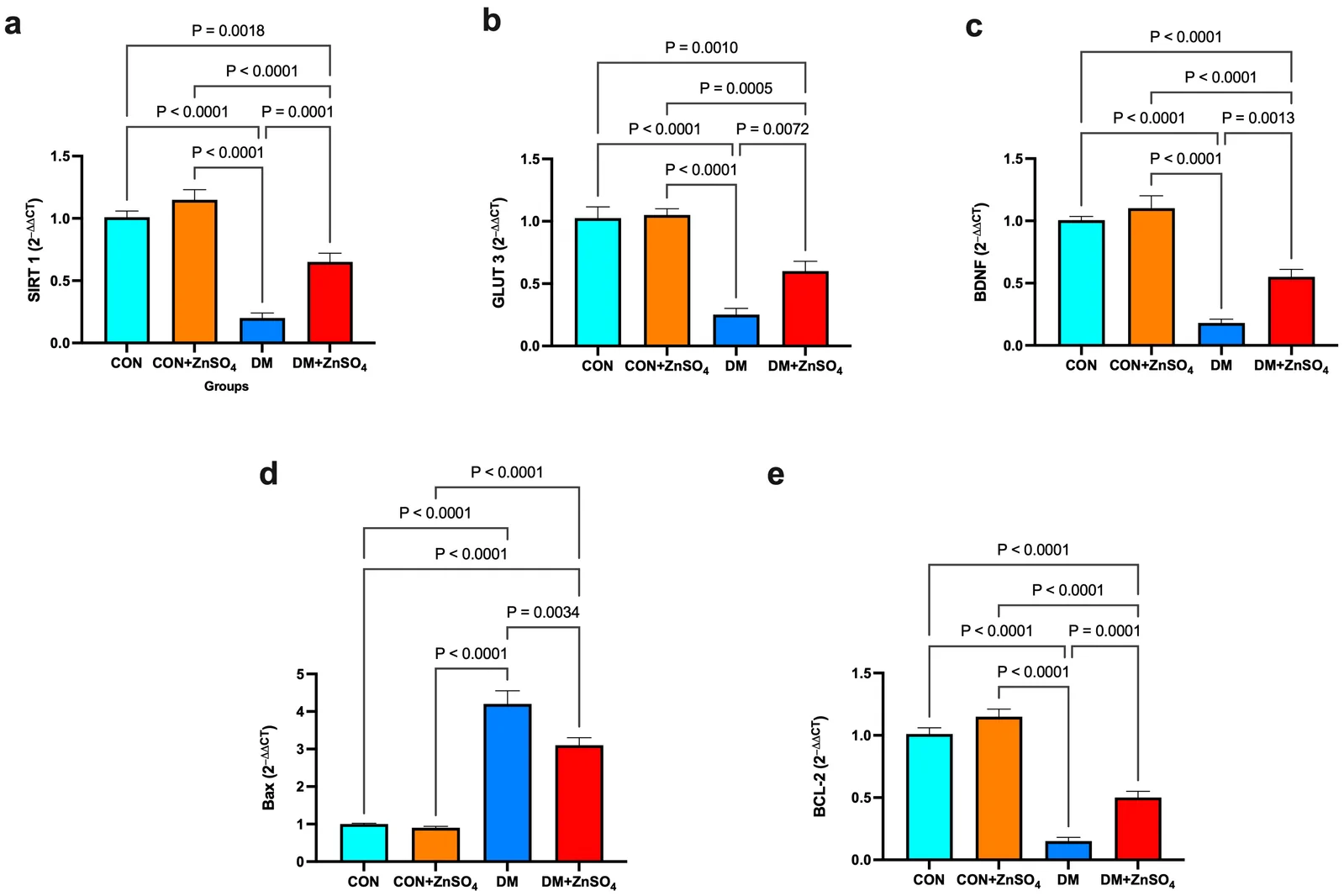
Effects of ZnSO_4_ treatment on Hippocampal Gene Expression Profiles in an Aged T2DM Rat Model. (**a**) The cellular stress response regulator SIRT-1, (**b**) the neuronal glucose transporter GLUT3, (**c**) the synaptic plasticity factor BDNF, (**d**) the pro-apoptotic marker Bax, and (**e**) the anti-apoptotic marker Bcl-2 mRNA expression levels. The different experimental groups are distinguished using a highly contrasting color scheme (CON: turquoise, CON+*ZnSO*_4_: orange, DM: blue, and DM+*ZnSO*4: red). The exact *p*-values indicating statistically significant differences between groups are shown directly on the respective figure panels. Data are expressed as Mean ± Standard Error of the Mean (SEM) (*n* = 8).

It was found that SIRT-1 mRNA expression levels measured in hippocampal tissue homogenates were statistically significantly lower (*p* < 0.0001) in the DM group (0.20 ± 0.04) compared to the CON group (1.00 ± 0.05). In the DM + ZnSO_4_ group, which received ZnSO_4_ treatment, SIRT-1 expression (0.65 ± 0.07) was found to be significantly increased (*p* < 0.0001) compared to the untreated DM group. The expression levels in the CON + ZnSO_4_ group, which received only ZnSO_4_ (1.15 ± 0.08), were found to be statistically similar to those in the CON group (Figure 2a).

The DM group showed significantly lower (*p* < 0.0001) GLUT3 mRNA expression levels compared to the CON group (1.00 ± 0.06). In the DM + ZnSO_4_ group receiving ZnSO_4_ supplementation, this level (0.60 ± 0.08) was found to be significantly higher (*p* < 0.0001) compared to the DM group. The expression level in the CON + ZnSO_4_ group was similar to that of the CON group (Figure 2b).

When examining hippocampal BDNF mRNA expression levels, it was observed that values in the DM group showed a statistically significant decrease (*p* < 0.0001) compared to the CON group. It was determined that ZnSO_4_ treatment administered in the DM + ZnSO_4_ group (0.55 ± 0.06) significantly increased (*p* < 0.0001) BDNF expression compared to the DM group. The expression levels in the CON + ZnSO_4_ group were found to be consistent with those of the CON group (Figure 2c).

It was found that the expression of Bax mRNA, a pro-apoptotic gene, was statistically significantly higher (*p* < 0.0001) in the DM group compared to the CON group. In the DM + ZnSO_4_ group, which received ZnSO_4_ supplementation, Bax expression levels were found to be significantly lower (*p* < 0.0001) than those in the untreated DM group. The values for the CON + ZnSO_4_ group (0.90 ± 0.04) were found to be similar to those of the control group (Figure 2d).

The anti-apoptotic Bcl-2 mRNA expression level was found to be significantly lower (*p* < 0.0001) in the DM group (0.15 ± 0.03) compared to the CON group. In contrast, Bcl-2 expression was significantly elevated (0.50 ± 0.05) (*p* < 0.0001) in the DM + ZnSO_4_ group treated with ZnSO_4_ compared to the DM group. In the CON + ZnSO_4_ group, this value was found to be statistically similar to that of the CON group, as shown in Figure 2e.

### 2.4. Histopathological and Immunohistochemical Findings

**Figure 3:**
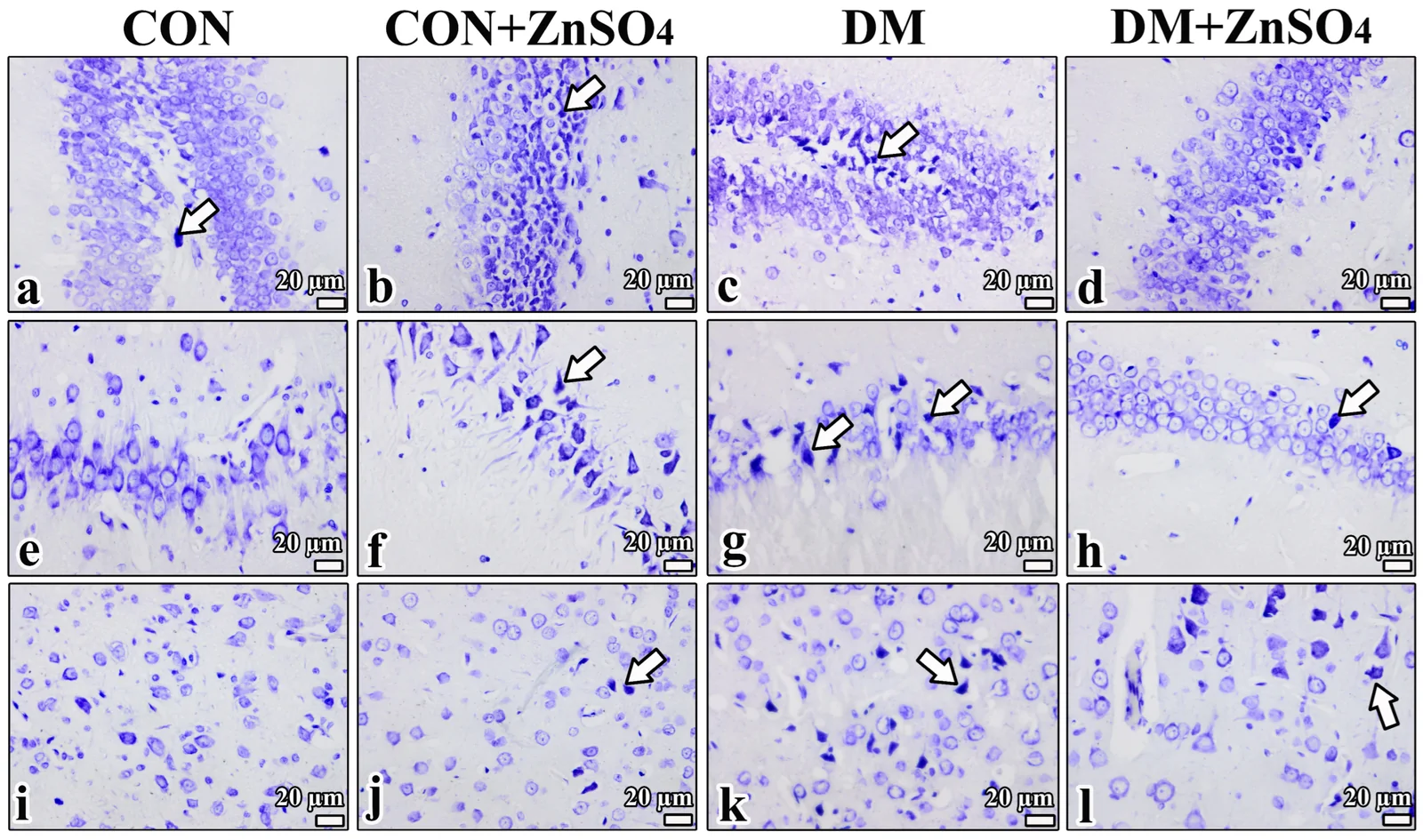
(**a**–**l**) Images obtained from sections stained with cresyl violet from the different groups. In all images, degenerate neurons are indicated by white arrows. (**a**–**d**) Images of the dentate gyrus (DG) are shown. (**b**) In the CON+ZnSO_4_ group, darkly stained structures presumed to be degenerate cells are observed in the granular and subgranular layers. Neuronal structures with cytoplasmic vacuolization and pyknotic nuclei are frequently seen. (**c**) In the DM group, densely darkly stained neurons in the subgranular layer are thought to be undergoing cell death. (**a**,**d**,**e**,**h**) In the CON and DM+ZnSO_4_ groups, healthy neurons are observed in the DG and cornu ammonis (CA) regions. Furthermore, no abnormalities are observed in the neuronal arrangement architecture. All cells are closely packed. (**f**,**g**) In the images of the CON+ZnSO_4_ and DM groups, disruptions in neuronal arrangement and an increase in the number of degenerate neurons are observed. Pycnotic nuclei are frequently observed, particularly in the DM group. (**j**–**l**) In the cortical images of the groups, degenerate, darkly stained neurons are noticeable in the CON+ZnSO_4_, DM, and DM+ZnSO_4_ groups. On the other hand, cytoplasmic vacuolization and nuclear fragmentation are commonly observed in neurons in the DM group. Scale bars: 20 µm.

**Figure 4:**
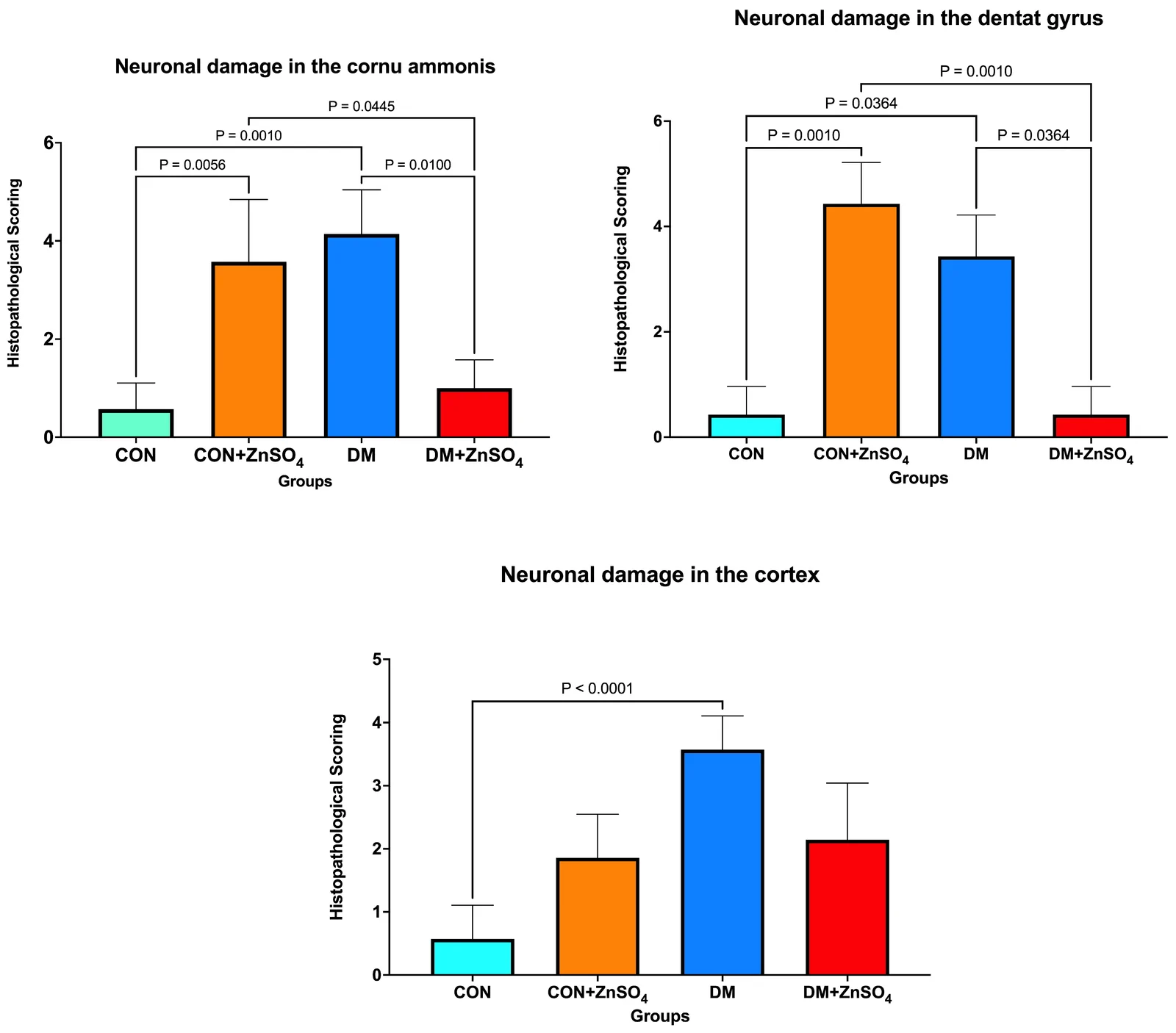
Histopathological scoring graphs of neuronal degeneration in the dentate gyrus and Ammon’s horn regions of the hippocampus and the cortical region for all groups. Individual experimental groups are represented by distinct, high-contrast color-coded bars (CON: turquoise, CON+*ZnSO*_4_: orange, DM: blue, and DM+*ZnSO*_4_: red) to ensure clear distinction. A *p*-value < 0.05 is considered statistically significant.

Light microscopic examination of the dentate gyrus (DG), Ammon’s horn (CA), and cortical structures of the hippocampus in all groups revealed significant degeneration in neurons of the CON+ZnSO_4_ and DM groups. Specifically, the degeneration, which was widespread in the subgranular region of the DG—where neurogenesis is intense in the DM group—was more pronounced in both the granular and subgranular regions of the CON+ZnSO_4_ group, with noticeable cytoplasmic vacuolization. The vast majority of neurons in the DG and CA regions of both the CON and DM+ZnSO_4_ groups exhibited normal morphology. Nuclear fragmentation and densely stained nuclear structures were also observed in the pyramidal neurons of the brain cortex in the DM group (Figure 3 and Figure 4). When neuronal damage in the cornu ammonis was evaluated, a significant increase was observed in the CON+ZnSO_4_ group compared to the DM+ZnSO_4_ and CON groups (*p* < 0.05, *p* < 0.01). A significant increase was also observed in the DM group compared to the DM+ZnSO_4_ and CON groups (*p* < 0.05). Similarly, neuronal damage in the dentate gyrus was found to be greater in the CON+ZnSO_4_ group compared to the DM+ZnSO_4_ and CON groups (*p* < 0.05). Likewise, an increase in the scoring of damaged neurons was observed in the DM group compared to the DM+ZnSO_4_ and CON groups (*p* < 0.05).

When neurons in the cortical region were examined, a higher density of neuronal damage was observed in the DM group compared to the other groups (*p* < 0.0001) (Figure 4).

**Figure 5:**
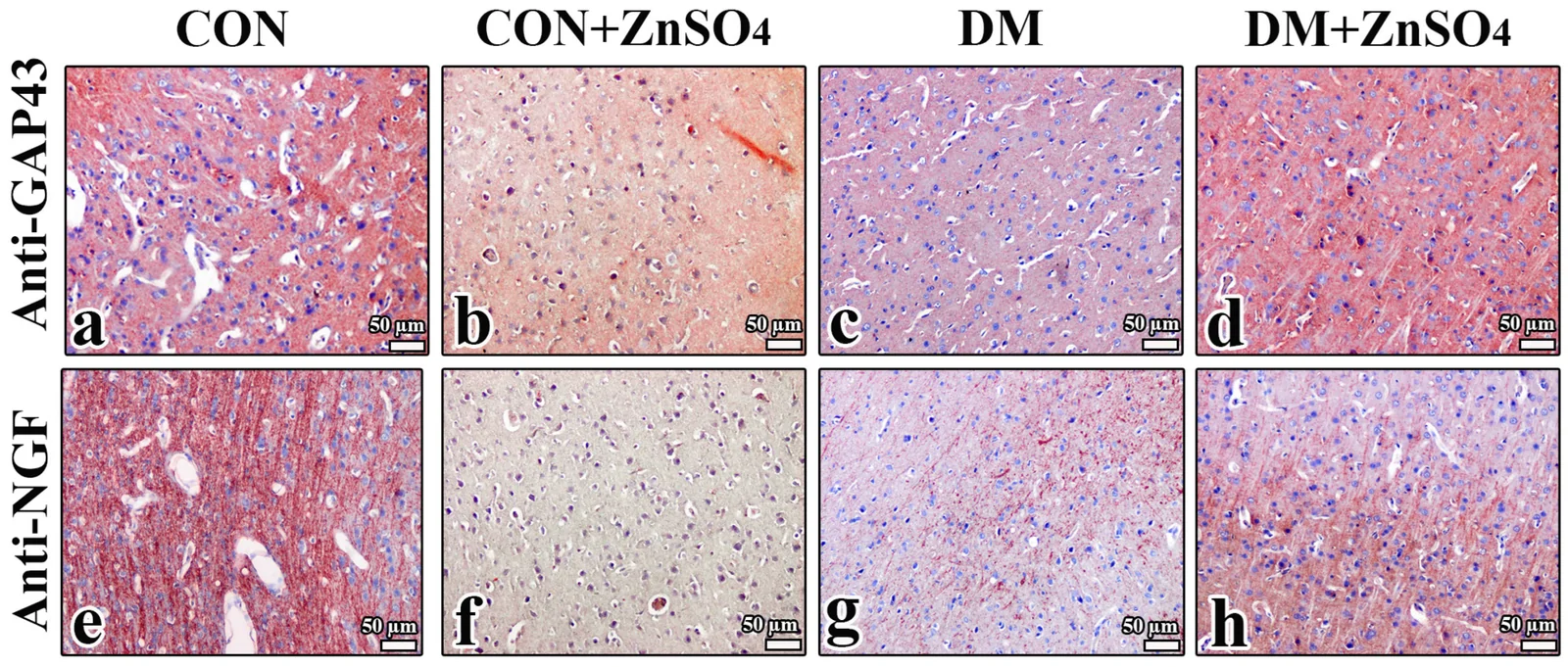
(**a**–**h**) The intensity of anti-GAP43 and anti-NGF expression in the cerebral cortex is shown for all groups. (**b**,**c**,**f**,**g**) Low-intensity immunoreactivity is observed in the CON+ZnSO_4_ and DM groups. Mayer’s hematoxylin was used for counterstaining. Positively stained areas appear brown-red. Scale bars: 50 µm.

**Figure 6:**
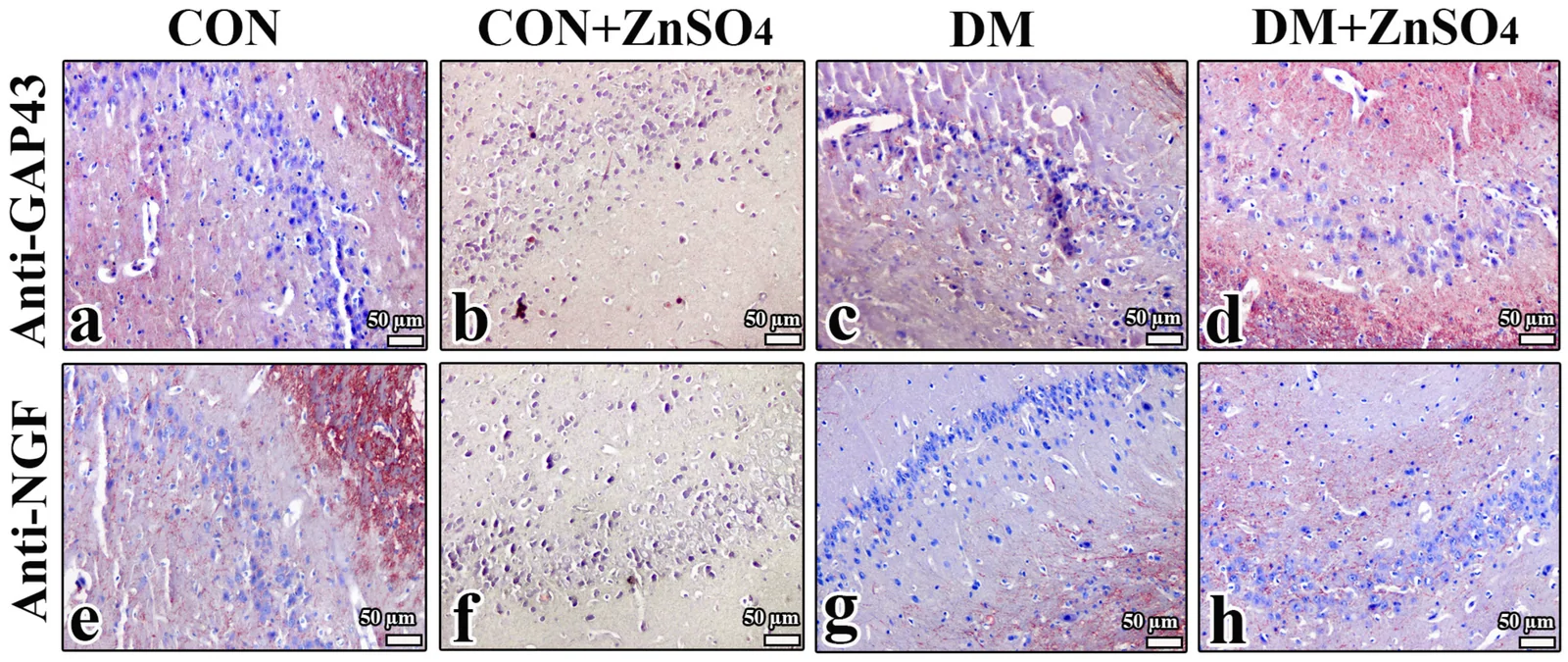
(**a**–**h**) The intensity of anti-GAP43 and anti-NGF expression in the hippocampus is assessed for all groups. (**b**,**c**,**f**,**g**) Low-intensity immunoreactivity is observed in the CON+ZnSO_4_ and DM groups. (**d**,**h**) However, anti-GAP43 staining is observed to be intense in both the control and DM+ZnSO_4_ groups. (**c**,**d**,**f**,**g**) Nevertheless, the anti-NGF staining in the DM+ZnSO_4_ group is noticeably similar to that of the DM group. Mayer’s hematoxylin was used for counterstaining. Positively stained areas are shown as brown-red. Scale bars: 50 µm.

**Figure 7:**
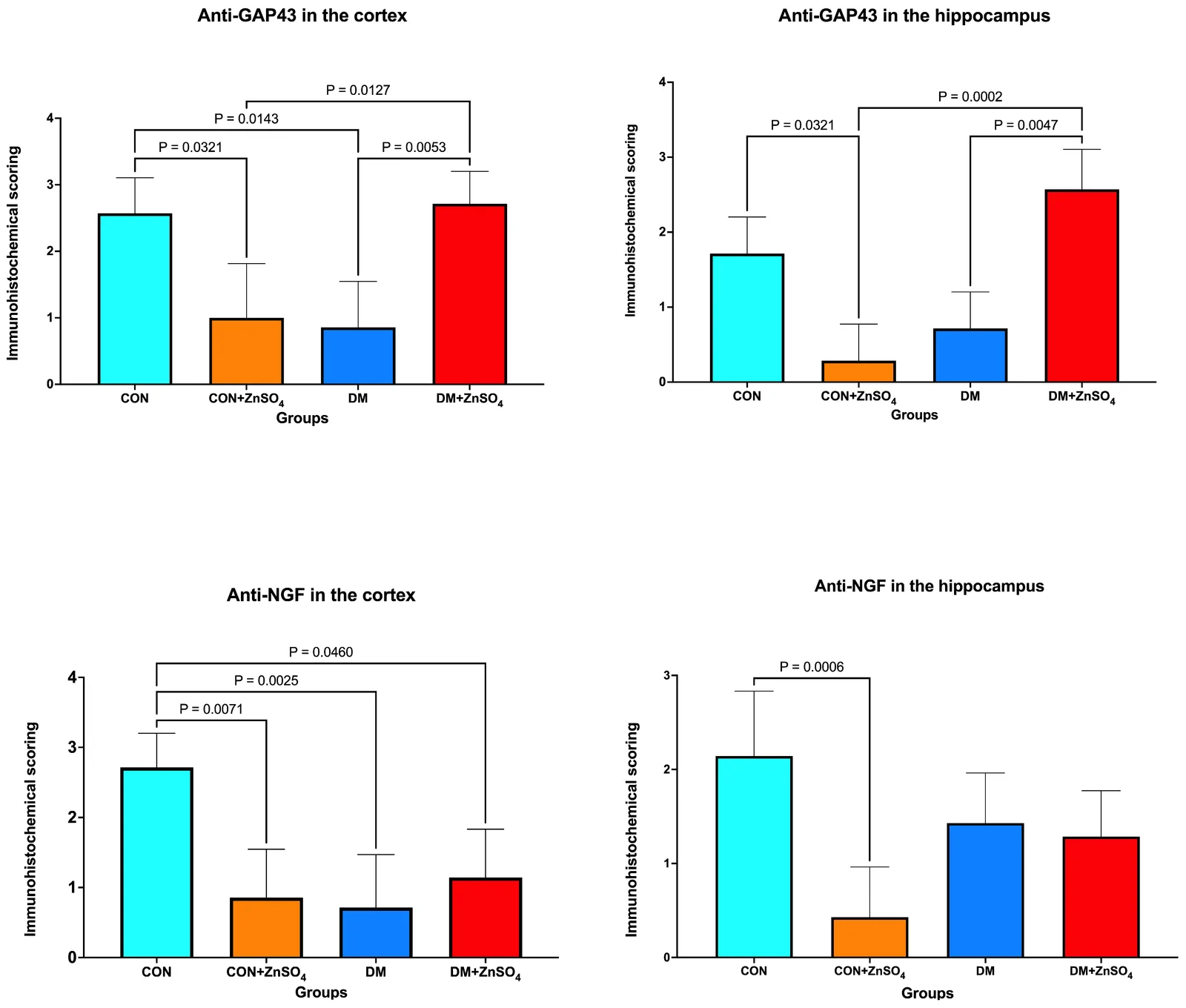
Immunohistochemical scoring graphs for anti-GAP43 and anti-NGF in the hippocampus and cortex regions for all groups. Individual experimental groups are represented by distinct, high-contrast color-coded bars (CON: turquoise, CON+*ZnSO*_4_: orange, DM: blue, and DM+*ZnSO*_4_: red) to ensure clear distinction. A *p*-value < 0.05 is considered statistically significant.

When evaluated for anti-GAP43 immunoreactivity, lower expression was observed in the cortex of the DM and CON+ZnSO_4_ groups compared to the CON and DM+ZnSO_4_ groups (*p* < 0.05), and similarly, significantly lower expression was observed in the hippocampus of the DM group compared to the DM+ZnSO_4_ group (*p* < 0.01). However, no difference was observed between the CON and DM groups (*p* > 0.05). Nevertheless, anti-GAP43 expression was lower in intensity in the CON+ZnSO_4_ group compared to the CON and DM+ZnSO_4_ groups (*p* < 0.05, *p* > 0.01). When the groups were evaluated for anti-NGF immunoreactivity, lower staining intensity was observed in the cortex across all experimental groups compared to the CON group (*p* < 0.05 and *p* < 0.01). Apart from this, no differences were detected between the other groups. In the hippocampus, a significantly greater reduction was observed, particularly in the CON+ZnSO_4_ group, compared to the CON group (*p* < 0.01). No difference was found between the other groups (*p* > 0.05) (Figure 5, Figure 6 and Figure 7).

**Figure 8:**
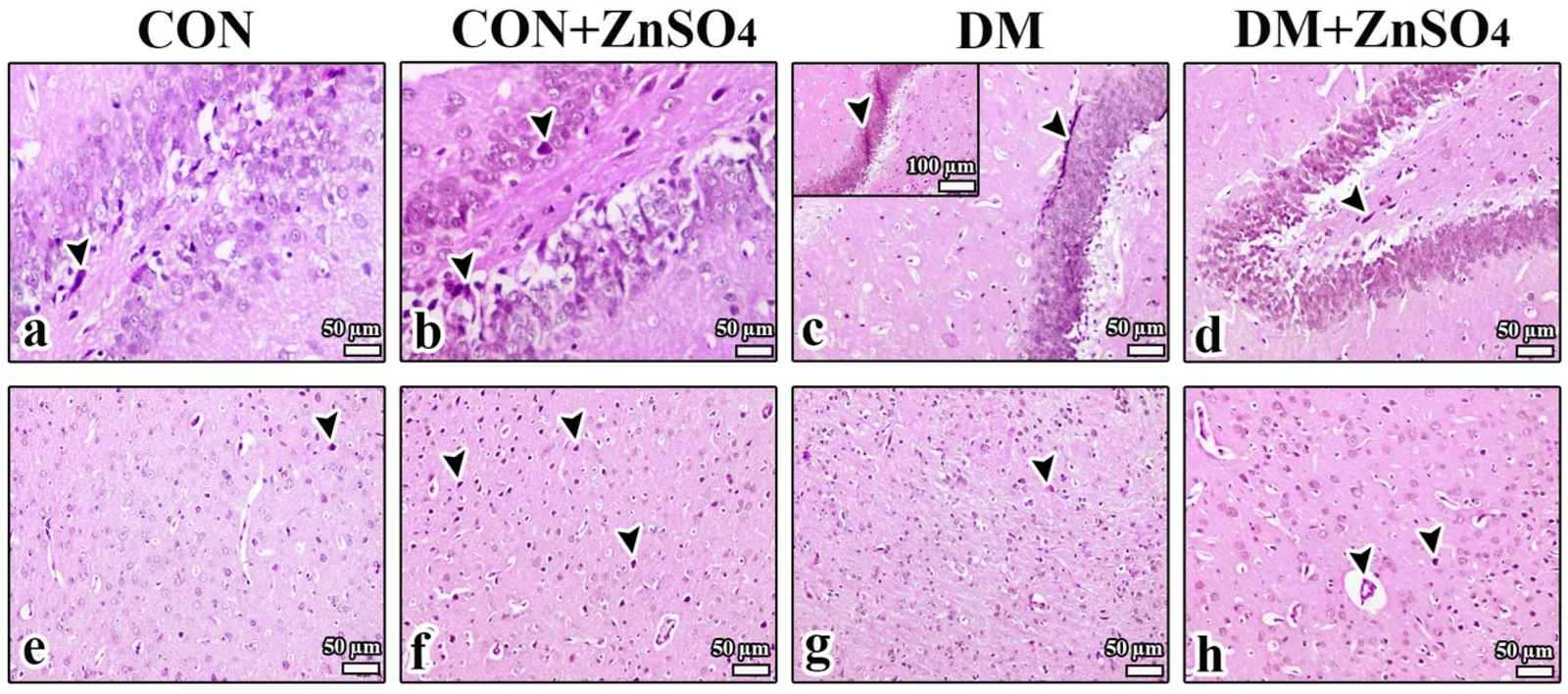
Representative photomicrographs of PAS staining in the hippocampus and cerebral cortex across all experimental groups, demonstrating glycogen deposition and vascular/neuronal pathologies. (**a**–**d**) Dentate gyrus (DG) region of the hippocampus: (**a**) CON group showing healthy tissue architecture with negligible PAS staining; (**b**) CON+ZnSO_4_ group displaying significant PAS-positivity in the granular and subgranular layers, suggesting altered glycogen management; (**c**) DM group exhibiting intense and widespread PAS-positivity in the DG, indicating diabetes-induced vascular and tissue degeneration; (**d**) DM+ZnSO_4_ group showing markedly reduced PAS staining intensity in the DG, with only sporadic PAS-positive neurons localized in the CA4 region. (**e**–**h**) Cerebral cortex region: (**e**) CON group displaying normal cortical histology with only a small number of isolated PAS-positive degenerate neurons; (**f**) CON+ZnSO_4_ group showing localized PAS-positivity within neurons and vessel walls; (**g**) DM group exhibiting prominent, concentrated PAS-positivity in both degenerate pyramidal neurons and microvascular walls, reflecting diabetic microvasculopathy; (**h**) DM+ZnSO_4_ group showing attenuated and localized PAS-positivity in cortical neurons and vascular structures.Arrowheads indicate PAS-positive neurons and vascular structures. Scale bars: 50 µm.

Periodic Acid-Schiff (PAS) positivity was evaluated in all groups, and significant positivity was observed in the dentate gyrus (DG) region in the CON+ZnSO_4_ and DM groups. In the DM+ZnSO_4_ group, positivity in the DG region was not very intense. However, PAS-positive neurons were found sporadically in the CA4 region of this group. Upon examination of cortical sections from all groups, it was noted that PAS positivity was concentrated in neurons and present in the vessel wall in the DM, CON+ZnSO_4_, and DM+ZnSO_4_ groups. PAS positivity was observed in a small number of degenerate neurons in the CON group (Figure 8).

## 3. Discussion

This study examined the modulatory effects of ZnSO_4_ administration on the systemic lipid profile and neurodegenerative processes in brain tissue in an aged model of T2DM induced by a high-fat diet and low-dose streptozotocin. Biochemical and molecular analyses were performed on hippocampal tissue to provide a comprehensive map of T2D-associated cognitive and structural brain damage, including cellular aging, synaptic plasticity, oxidative stress, and apoptosis. The histopathological aspect of diabetic neurodegeneration and zinc’s protective effects on structure was also assessed at the cellular level, extending beyond the hippocampus to the cerebral cortex, which is the primary center for higher cognitive functions and information integration. The results show that zinc substantially restores impaired systemic metabolism in a diabetic environment and repairs neuro-molecular damage at both the hippocampal and cortical levels.

Observations of changes in serum lipid profiles—a key indicator of metabolic disorders—showed that total cholesterol, triglyceride, and LDL levels significantly increased in diabetic rats, whereas protective HDL levels significantly decreased. These findings are in line with existing research using the HFD-STZ model, which has shown that insulin resistance and high blood sugar disrupt normal lipid metabolism, resulting in abnormal levels of lipids in the blood [20]. Our study found that the lipid profile in the diabetic group receiving ZnSO_4_ treatment improved significantly towards the levels of the control group. Research indicates that zinc functions as a cofactor for enzymes involved in lipid metabolism regulation, enhances insulin sensitivity by activating cellular energy sensors like the AMPK pathway, and mitigates systemic stress resulting from dyslipidemia [21,22].

Systemic dyslipidemia and chronic hyperglycemia disrupt cellular redox balance by increasing reactive oxygen species (ROS) production in brain tissue [8,23]. Our study found that levels of MDA—a marker of cellular membrane damage—were higher in the hippocampal tissues of diabetic rats, whereas levels of the brain’s main antioxidant, reduced glutathione, were lower. The diminished capacity to counter oxidative stress facilitates the acceleration of the neurodegenerative process seen in diabetic encephalopathy. Our study found that ZnSO_4_ treatment led to a decrease in MDA levels and an increase in GSH levels, which confirms zinc’s antioxidant properties in neuronal tissue. Liu et al. (2014) also found that intraperitoneal administration of zinc to STZ-induced diabetic rats led to a significant decrease in MDA levels in neural tissues and the activation of endogenous antioxidant mechanisms [10]. Zinc, a redox-inert trace element, is understood to protect hippocampal cells from free radical damage by supporting glutathione synthesis and activity [19].

Direct oxidative stress suppresses cellular pathways that regulate energy metabolism and the stress response. In our study, the mRNA expression of the SIRT-1 enzyme, which protects cells from oxidative stress, was significantly reduced in the diabetic group but was upregulated by ZnSO_4_ treatment. Meng et al. (2020) reported in detail that chronic hyperglycemia reduces SIRT-1 expression; conversely, SIRT-1 activation counteracts cellular stress by stimulating antioxidant targets [8]. The diabetic group also showed a reduction in the expression of the GLUT3 transporter, which is involved in neuronal glucose uptake, and BDNF, which is responsible for synaptic plasticity. Essential elements like zinc protect the expression of glucose transporters (GLUT3) and BDNF production in the brain [7]. Choi et al. (2020) also demonstrated that synaptic zinc in the hippocampus plays a crucial role in maintaining BDNF levels, which are essential for neurogenesis and learning [19]. Our findings show that ZnSO_4_ supplementation restores neuronal energy starvation (GLUT3) and synaptic impairment (BDNF) through SIRT-1 activation.

Oxidative stress and decreased neurotrophic support induce apoptosis in neurons. Our study found that the diabetic group showed an increase in Bax expression and a decrease in Bcl-2 expression. STZ-induced hyperglycemia directly triggers neuronal apoptosis through accelerated degenerative processes in brain cells, as histopathologically documented [24]. In our study, the ZnSO_4_ treatment reduced cellular death by altering the Bax/Bcl-2 ratio to promote neuronal survival. Cavalcanti et al. (2019) discovered that ZnSO_4_ treatment in diabetic rat models exhibited neuroprotective and anti-apoptotic effects, achieved by decreasing the number of ischemic/degenerated neurons in brain tissue [18].

While preclinical models demonstrate robust cellular benefits, the translational significance of zinc supplementation is heavily supported by human clinical studies, although it operates as a double-edged sword depending on the dosage. In clinical trials, moderate zinc supplementation (typically 15 mg/day) has been shown to improve glycemic biomarkers, reducing fasting plasma glucose (FPG) and HbA1c by 5–15%, particularly when administered as a metabolic co-adjuvant in patients with T2DM. For instance, a randomized, double-blind, placebo-controlled trial in zinc-deficient diabetic patients on hemodialysis demonstrated that oral zinc sulfate supplementation significantly reduces systemic inflammatory markers, including C-reactive protein (CRP), and improves overall metabolic profiles. Furthermore, in critically ill patients with severe closed head trauma, clinical studies have confirmed that zinc supplementation accelerates neurological recovery, significantly improves Glasgow Coma Scale (GCS) ratings, reduces organ failure indices (SOFA scores), and dampens systemic inflammatory responses (reducing CRP, erythrocyte sedimentation rate, and white blood cell count). In healthy human subjects, zinc supplementation has also been documented to suppress lipid peroxidation and oxidative stress markers, such as plasma malondialdehyde (MDA) and 8-hydroxydeoxyguanine. However, clinical zinc administration must be carefully managed to avoid potential toxic effects. At the cellular level, excessively high concentrations of mobile zinc can trigger potent neurotoxic cascades, promoting cell death through NADPH oxidase overactivation and mitochondrial reactive oxygen species (ROS) generation during acute insults like stroke or severe hypoglycemia. Chronically, prolonged high-dose zinc supplementation interferes with the intestinal absorption of copper, leading to zinc-induced copper deficiency. This condition can manifest clinically as severe hematological anomalies, such as refractory anemia and neutropenia, as well as progressive neurological symptoms and sensory ataxia. Therefore, identifying the therapeutic window and optimizing tissue-specific zinc homeostasis is of paramount clinical importance for its safe integration into standard diabetes care.

Our histopathological data examining cellular integrity directly align with biochemical and molecular findings of antioxidant and anti-apoptotic effects. The physical signs of the neurodegenerative process related to diabetes extend beyond the hippocampus—the primary site of learning and memory—to profoundly affect the cerebral cortex, which is responsible for higher-level brain functions [5,24]. Our study’s histopathological and immunohistochemical findings, specifically for GAP43 and NGF, show that ZnSO_4_ treatment protects cells and molecules by activating the SIRT-1/BDNF pathway and significantly reduces neuronal degeneration and vascular pathologies (PAS positivity) in both the dentate gyrus (DG) and cornu ammonis (CA) regions of the hippocampus, as well as in cortical tissue.

When histopathological and immunohistochemical data were evaluated, it was observed that neurons belonging to the CON group had distinct nuclear and nucleolar boundaries. Neurons in this group were found to stain euchromatically and to be healthy. Degeneration in the DG and CA regions of the hippocampus in the DM and CON+ZnSO_4_ groups indicates neurotoxicity. In the CON+ZnSO_4_ group, the intense degeneration observed in the DG was seen in the subgranular and granular layers. In this case, it can be considered that ZnSO_4_ application negatively affects neurogenesis. Cytoplasmic vacuolization observed in the DM and CON+ZnSO_4_ groups may indicate cellular stress. When PAS staining was evaluated, very few positive results were noted in the DG of healthy rats. This may be due to glycoproteins in the structure of senile plaques that appear with age. It is also known that PAS positivity in the hippocampus is significantly increased with diabetes. The positive results observed in the brain tissue indicate vascular pathology caused by DM. Zinc plays a crucial role in glucose homeostasis [25]. In neurons, the role of zinc is twofold. Under dominant stress, the effect of zinc on glycogen management can change. It is known that excess zinc in the brain can have effects similar to Alzheimer’s disease. It has been found that zinc administration disrupts the structure of tau protein [26]. Histological images suggest that zinc exposure, particularly in the hippocampus and cortex, disrupts glucose homeostasis in the brain and increases PAS positivity. The effects of DM on glucose homeostasis in the brain are similar to the effects of zinc. It is known that GAP43 expression is reduced in the brain in DM. This is significant evidence that DM reduces neuroplasticity in the brain [27]. Decreased levels of GAP-43 protein, a key indicator for memory formation and axon regeneration, may be one of the underlying causes of cognitive dysfunction in DM. Our study shows that this condition can be compensated for with ZnSO_4_ treatment. A previous study suggested that zinc oxide nanoparticles induce oxidative stress in the brain and also reduce neuronal regeneration capacity by disrupting the GAP-43 protein [28]. Our immunohistochemical findings are consistent with the literature. Our data show that NGF is also expressed at lower intensity with zinc administration. Similarly, decreased NGF expression in diabetes can be considered evidence of DM’s suppressive role on neurogenesis. Zinc is known to play a role in the regulation of various cell division growth factors, such as NGF. NGF plays a role in the development of neuronal populations and neuronal plasticity in the peripheral and central nervous systems [19]. On the other hand, excess zinc ions can disrupt the NGF protein. Zinc, which can alter the structure of NGF, may thus prevent NGF2 from protecting neurons [29]. Our study suggests that zinc increases GAP43 reactivity in a diabetic setting and likely exerts a neuroprotective effect through this mechanism. However, further research is needed to investigate the mechanisms underlying zinc’s therapeutic effect in a diabetic environment. The lack of support for the observed plasticity effect in the hippocampus through behavioral tests is a limiting factor in our study. Although functional cognitive evaluations were not actively performed in this specific cohort, the therapeutic potential of zinc in reversing diabetic and neurodegenerative cognitive impairments is strongly corroborated by previous functional evidence from our research group and the wider literature. In a highly relevant sporadic Alzheimer’s-like model in Wistar rats, we previously demonstrated that daily treatment with zinc sulfate (5 mg/kg/day, i.p.) completely rescued severe spatial learning and memory deficits in the Morris Water Maze task, as shown by restored escape latencies and target quadrant exploration times back to healthy sham levels. Crucially, this behavioral rescue was directly mirrored by the molecular upregulation of hippocampal BDNF and SIRT1, validating the pathophysiological translational value of the SIRT-1/BDNF upregulation observed in our current Type 2 diabetic rats. This is further supported by Cavalcanti et al. (2019) [18], who demonstrated that zinc sulfate supplementation (15 mg/kg/day) exerts potent anxiolytic-like effects and counters behavioral despair in diabetic rodent models. Consequently, while the absence of active behavioral assays in the current study is acknowledged as a limitation, our molecular findings are firmly aligned with established functional datasets demonstrating zinc’s capacity to restore cognitive and emotional homeostasis. Several limitations of the present study must be acknowledged. First, regarding the biological rationale of our treatment, ZnSO_4_ was administered via intraperitoneal injection, bypassing gastrointestinal absorption. Upon systemic entry as free Zn^2+^, its penetration into the brain strictly depends on tightly regulated blood–brain barrier (BBB) transport systems. While we selected the inorganic ZnSO_4_ salt to maintain methodological consistency with our previously validated protocols [17] and its widespread use as a standard reference compound in preclinical models of diabetic encephalopathy and neuropathy [10,18], we acknowledge that organic zinc formulations generally exhibit higher central bioavailability than inorganic zinc salts. Consequently, utilizing highly bioavailable organic zinc compounds could potentially yield amplified therapeutic profiles, and the lack of a direct comparison between inorganic and organic zinc formulations is a limitation of this study. Second, we did not directly measure zinc concentrations in the serum or brain tissues. Although the systemic and molecular improvements observed in the treatment groups strongly imply the effective uptake and utilization of the supplemented zinc, the lack of direct quantification of zinc bioavailability remains a limitation. Future investigations should incorporate direct analytical measurements of tissue zinc levels to establish a more definitive correlation between zinc supplementation and its neuroprotective effects. Finally, the present study was conducted exclusively on aged female rats without a parallel male cohort or active estrous cycle monitoring. While focusing on a single sex limits the generalizability of our findings, this selection was highly intentional and translationally relevant. In clinical settings, females with Type 2 diabetes exhibit a significantly higher risk for accelerated cognitive decline and mild cognitive impairment than males, a disparity driven by the postmenopausal decline in estrogen-mediated neuroprotective pathways. By utilizing 18-month-old rats, which are in a state of completed reproductive senescence (acyclic/postmenopausal state), we modeled the clinical postmenopausal demographic. Consequently, active estrous cycle monitoring (such as daily vaginal smears) was physiologically redundant, as these aged animals no longer undergo regular follicular phases or cyclic sex hormone fluctuations. The chronic depletion of ovarian sex hormones (mainly estrogen and progesterone) in these aged acyclic animals removes a critical neuroprotective, antioxidant, and insulin-sensitizing shield, thereby directly exacerbating brain vulnerability to hyperglycemia-induced oxidative damage, mitochondrial dysfunction, and neuroinflammation. Additionally, a comparative male cohort was not included in strict adherence to the ethical principle of Reduction (3Rs)—as the baseline metabolic and therapeutic dynamics of zinc in male diabetic rodents are already widely documented in the literature. Nonetheless, future sex-comparative investigations across different age groups are warranted to comprehensively map these dimorphic pathways. While our preclinical model focused on aged female rats to represent a postmenopausal state characterized by heightened susceptibility to diabetic encephalopathy, the lack of a parallel male cohort remains an acknowledged limitation. In clinical settings, females with Type 2 diabetes exhibit a significantly higher risk for accelerated cognitive decline and mild cognitive impairment than their male counterparts, a disparity largely attributed to the postmenopausal decline in estrogen-mediated neuroprotective pathways. Consequently, focusing specifically on aged females allowed us to characterize these understudied and highly vulnerable molecular dynamics. Nonetheless, direct sex-comparative studies are essential to fully map the dimorphic pathophysiological pathways and determine whether the therapeutic window of zinc differs between sexes.

## 4. Materials and Methods

### 4.1. Experimental Animals and Preparation Phase

This study adhered to national and international guidelines for animal welfare and used the ARRIVE guidelines to ensure transparent and reliable reporting of in vivo animal research. All experimental protocols and procedures were approved by the Local Ethics Committee of KTO Karatay University Faculty of Medicine (Approval No: 2026/051, 30 April 2026).

In the study, aged female rats (18 months old) were selected because recent clinical evidence identifies the female sex as a major risk factor and the most vulnerable demographic profile for Type 2 diabetes (T2DM)-associated cognitive decline (diabetic encephalopathy) [30,31,32]. Given their advanced age (18 months old), the female rats used in this study were in a state of completed reproductive senescence (acyclic/postmenopausal state). Therefore, estrous cycle monitoring (such as daily vaginal smears) was not performed, as these animals no longer undergo regular follicular phases or cyclic sex hormone fluctuations. The experimental animals were housed under controlled standard laboratory conditions with a 12 h light/12 h dark cycle, a room temperature of 22 ± 2 °C, and a relative humidity of 55 ± 5%. The animals had free (ad libitum) access to standard laboratory pellet feed and clean drinking water until the start of the dietary and experimental modeling phase. All rats were allowed a one-week acclimatization period prior to the start of the experiments to minimize stress caused by experimental procedures and to allow them to adapt to the laboratory environment.

### 4.2. Formation of Experimental Groups and Drug Administration

Following the one-week acclimatization period, the animals were randomized based on their initial body weights and blood glucose levels and divided into the four main experimental groups detailed below (*n* = 8 per group, total *n* = 32). The sample size was determined through an a priori power analysis (using G*Power software, version 3.1.9.7) designed to achieve a statistical power of at least 80% (1-β = 0.80) with an alpha error level of 0.05, based on anticipated effect sizes from similar prior studies evaluating neuroprotective and biochemical parameters in diabetic models. This sample size ensures adequate statistical power while strictly adhering to the 3Rs (Reduction) principle of animal ethics:

Group 1—Control (CON): Comprises healthy, aged female rats fed a standard laboratory pellet diet (NPD) throughout the experimental period. This group’s regimen included a single intraperitoneal dose of 0.1 M citrate buffer (pH 4.4), in place of STZ, solely as a solvent, on the day of diabetes induction exactly as done by Mehta and Banerjee (2017) [5,33]. Normal saline was administered intraperitoneally on a daily basis. During the final 4 weeks of the experiment, the treatment phase was in effect.

Group 2—Control + Zinc Sulfate (CON+ZnSO_4_): This group comprises healthy rats fed a standard diet and administered citrate buffer instead of STZ by injection. Animals in this group received 10 mg/kg ZnSO_4_ via intraperitoneal injection. For the final 4 weeks of the experiment, the route was followed daily [10,33].

Group 3—Diabetes Mellitus (DM): Animals in this group were first fed a high-fat diet (HFD) for 4 weeks. Following a 12 h fast, a single dose of low-dose STZ (25 mg/kg), freshly dissolved in 0.1 M citrate buffer, was administered via intraperitoneal (i.p.) injection [5]. Animals with fasting blood glucose levels >250 mg/dL, as measured from the tail vein 72 h after STZ administration, were considered diabetic. Animals in this group received only normal saline via the i.p. route daily for 4 weeks following confirmation of diabetes (treatment phase).

**Figure 9:**
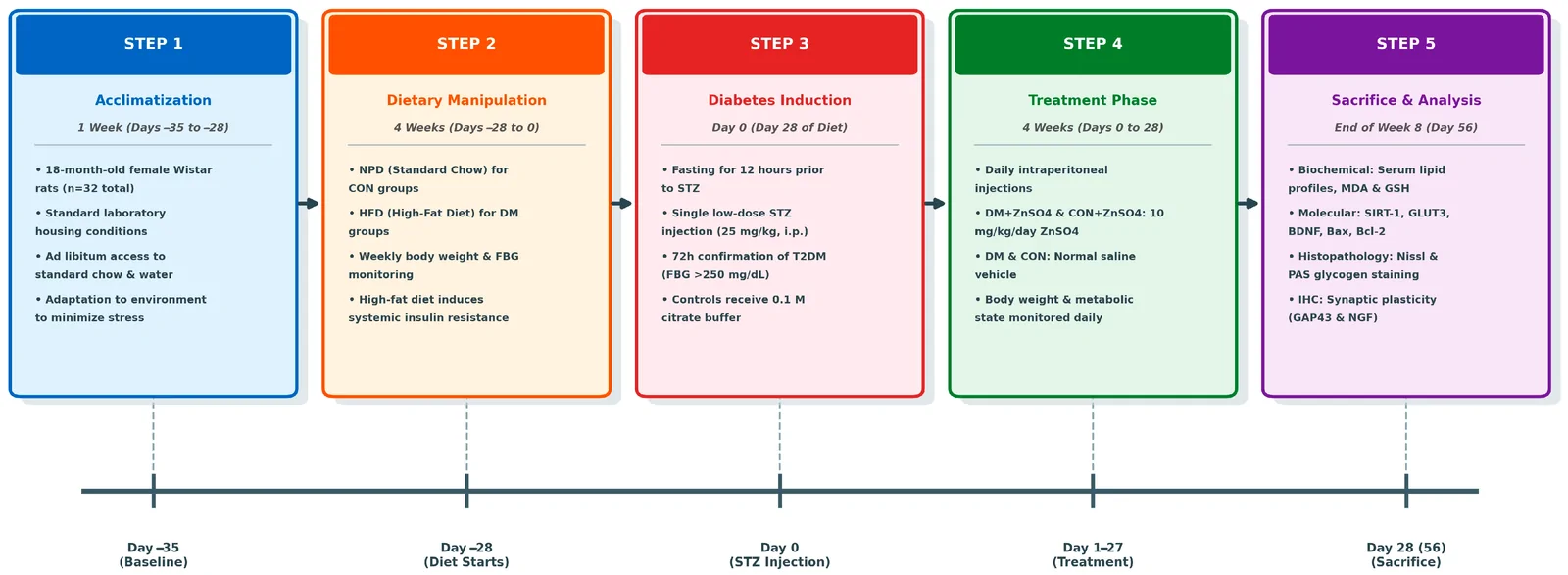
Schematic representation of the chronological experimental timeline and protocol steps in the aged Type 2 diabetic rat model. The arrows represent the sequential and chronological progression of the experimental protocol, guiding from the baseline acclimatization through high-fat diet (HFD) manipulation, streptozotocin (STZ) induction, the active treatment window, and final endpoint harvesting. Blue box (Step 1-Acclimatization): Represents the baseline stabilization phase where aged rats (18 months old) adapt to the laboratory environment to minimize standard physiological stress. Orange box (Step 2-Dietary Manipulation): Represents the diet-specific priming period where animals are fed standard chow (CON) or a high-fat diet (HFD) to establish baseline insulin resistance. Red box (Step 3-Diabetes Induction): Denotes the critical clinical intervention milestone involving fasting and a single low-dose STZ (25 mg/kg, i.p.) injection to selectively establish the Type 2 diabetic phenotype, followed by tail-vein glycemic verification (fasting blood glucose > 250 mg/dL). Green box (Step 4-Treatment Phase): Highlights the active therapeutic window where daily intraperitoneal injections of zinc sulfate (10 mg/kg/day) or saline vehicle are administered, alongside ongoing metabolic monitoring. Purple box (Step 5-Sacrifice & Analysis): Denotes the final experimental endpoint (at Day 28 of treatment/Day 56 total diet) dedicated to systemic lipid profiling, biochemical oxidative stress assays (MDA, GSH), hippocampal quantitative qPCR gene expression (SIRT-1, GLUT3, BDNF, Bax, Bcl-2), histopathology (Cresyl violet, PAS), and neuroplasticity immunohistochemistry (GAP43, NGF).

Group 4—Diabetes Mellitus + Zinc Sulfate (DM+ZnSO_4_): This group consists of rats that were made diabetic by following the same procedures as the T2DM group, which involved four weeks of a high-fat diet and a 25 mg/kg streptozotocin injection. Following confirmation of the diabetic state, ZnSO_4_ was administered i.p. at a dose of 10 mg/kg daily for 4 weeks to these animals to test its potential to prevent cellular damage and cognitive decline (Figure 9).

Animals in all groups were sacrificed after completing the 4-week ZnSO_4_ treatment protocol following the STZ injection, and hippocampal tissue analyses were performed. Rats were allocated to their respective cages using a computer-generated randomization sequence (using GraphPad Prism software, version 10.0, Boston, MA, USA). To minimize potential confounding microenvironmental factors, cage locations on the racks were systematically rotated weekly, and all downstream biochemical, molecular, and histopathological outcome assessments were performed in a randomized order.

### 4.3. Blood Sampling and Systemic Biochemical Analyses

Blood samples were collected immediately before the animals were sacrificed, after the completion of a 4-week treatment protocol. Blood samples were centrifuged at 15,000 rpm for 10 min to isolate plasma and serum. Serum levels of total cholesterol, triglycerides, HDL, and LDL were measured to assess the systemic dyslipidemia induced by HFD and STZ administration.

### 4.4. Biochemical Analyses of Hippocampal Tissue

Tissues from the hippocampus, isolated quickly after death and stored at −80 °C, were homogenized in suitable buffer solutions (pH 8.0 phosphate buffer containing 1% Triton X) and then centrifuged to obtain supernatants for the assessment of oxidative stress and cellular antioxidant capacity. Malondialdehyde (MDA), reflecting free radical damage and lipid peroxidation in the tissue, was measured using specific commercial ELISA kits (Elabscience, Wuhan, Hubei, Cat. No: E-EL-0060) in accordance with the manufacturer’s instructions. The levels of reduced glutathione (GSH), one of the brain’s most important endogenous antioxidant mechanisms, were also analyzed using specific ELISA kits (Elabscience, Cat. No: E-EL-0026). The biochemical analysis results were normalized according to protein concentration, with the total protein content in tissue homogenates determined by the Lowry method.

### 4.5. Molecular Analyses

Upon euthanizing the animals, brain samples were swiftly extracted to carefully isolate the hippocampal sections. Following the manufacturer’s instructions, total RNA extraction from the frozen hippocampus of each subject was performed utilizing a commercial isolation kit (Cat. No: BS88203, Bio Basic, Toronto, ON, Canada). The concentration and purity of the obtained RNA were spectrophotometrically evaluated via a microplate reader (Multiskan SkyHigh, Thermo Fisher, Waltham, MA, USA). Subsequently, complementary DNA (cDNA) was generated on a ProFlex PCR System (Thermo Fisher, USA) employing a High-Capacity cDNA Reverse Transcription Kit (Cat. No: 4368813, Thermo Fisher, Vilnius, Lithuania).

To assess the mRNA expression levels of target genes (SIRT-1, GLUT3, BDNF, Bax, and Bcl-2), quantitative real-time PCR (qPCR) was carried out. The amplification reactions were run in triplicate with a final volume of 20 µL, consisting of the FastStart Essential DNA Green Master mix (Cat. No: 06 402 712 001, Roche, Basel, Switzerland). All specific primers for the targeted genes were commercially provided by Oligomer (Çankaya, Turkey). To normalize the expression data, GAPDH served as the internal reference gene (Table 2). Finally, relative fold changes in gene expression were computed by applying the standard 2^−ΔΔCt^ method [34].

### 4.6. Tissue Processing, Sectioning, Staining, and Histopathological Scoring

After the experiment, brain tissue samples dissected from female rats were fixed in a mixture of 2% paraformaldehyde and 2% glutaraldehyde, and then 4 µm-thick sections were cut for histopathological evaluation and for immunohistochemical analysis using a microtome. For histopathological evaluation, the sections were stained with Cresyl violet after deparaffinization. At least 10 sections were evaluated for each animal, taken from different levels of the tissue in the sagittal plane, using a systematic random sampling interval (1/20), and histopathological scoring was performed in the dentate gyrus and cornu ammonis regions of the hippocampus and the cerebral cortex. Neurons in images obtained from sections were evaluated for hyperchromatic nuclei, pyknotic nuclei, cytoplasmic vacuolization, nuclear fragmentation, and neuron-deficient areas using a light microscope equipped with a camera attachment (Olympus, BX43, Center Valley, PA, USA) and the computer-assisted CellSens Entry microscopy software program (version 1.18; Olympus, Center Valley, PA, USA) at 40× and 20× magnifications. A semi-quantitative assessment of neuronal damage was conducted, and the following criteria were used for histopathological grading [35]:

(0): Healthy neuron

(1): Rare neuronal damage

(2): Moderate neuronal damage

(3): Common neuronal damage

(4): Widespread neuronal damage

(5): Severe neuronal damage

To eliminate investigator bias during outcome assessment, all histopathological evaluations and semi-quantitative scorings were performed in a double-blinded manner. Brain tissue sections were randomly assigned unique numeric codes by an independent researcher who was not involved in the scoring process, and the histopathologists remained completely blinded to the experimental group allocations until the entire quantitative data set was finalized.

### 4.7. Immunohistochemical Analyses

Immunohistochemical evaluation using anti-GAP43 (RRID AB_443303, dilution: 1:300, ab16053, Abcam, Waltham, MA, USA) and anti-NGF (RRID AB_2156475, dilution: 1:200, ab6199, Abcam, USA) primary antibodies was performed. Antigen retrieval was performed in a microwave oven using citrate buffer (pH 6.0): first at 850 watts for 3 min, then at 170 watts for 20 min. Immunohistochemical staining was performed according to the Mouse and Rabbit Specific HRP/AEC Detection IHC Kit (ab93705, Abcam, USA). Mayer’s hematoxylin was used for counterstaining. Immunohistochemical scoring was as follows: 0, no staining; 1, rare staining; 2, moderate staining. Sections were evaluated as follows: 3, good staining; 4, severe staining [36]. Images obtained from the sections were examined at 20× magnification on a light microscope (Olympus, BX43, Center Valley, PA, USA) equipped with a camera attachment and the computer-assisted CellSens Entry microscopy software program (Olympus, Center Valley, PA, USA).

Immunohistochemical staining intensity and positive cell counts were quantified under light microscopy by researchers completely blinded to the treatment groups, utilizing the same randomized coding system described for histopathological scoring.

### 4.8. Periodic Acid-Schiff (PAS) Staining

The Periodic Acid-Schiff (PAS) staining, which detects polysaccharides such as glycogen in brain tissue, was evaluated in the hippocampal and cortical structures. Tissues, initially deparaffinized in an incubator and then with xylene, were processed through a series of alcohol solutions. The tissues were then treated with periodic acid solution for 10 min. Sections treated with Schiff’s reagent at room temperature were washed with distilled water for 30 min. The sections were stained with Haemalum Mayer Hematoxylin for 5 min after being washed for 5–10 min and then examined under a light microscope. The Periodic Acid-Schiff Stain kit (Lot No. MK250115, Patolab, Esenler, Turkey) was used for staining.

### 4.9. Statistical Analysis

All data obtained from the study are presented as mean ± standard error of the mean (SEM). The suitability of the data for a normal distribution was assessed using the Shapiro–Wilk normality test. For statistical comparisons between groups, including multi-group comparisons such as molecular and biochemical analysis results, one-way ANOVA was applied. The Tukey test was preferred for post hoc analysis to identify differences between groups. Since the groups did not show a normal distribution in the analysis of immunohistochemical and histopathological scoring data, the Kruskal–Wallis test (post hoc: Dunn’s test) was used. The threshold for statistical significance was set at *p* < 0.05. All statistical calculations and the preparation of graphs were performed using GraphPad Prism software (version 10.0, Boston, MA, USA).

## 5. Conclusions

This study demonstrates that ZnSO_4_ administration significantly alleviates diabetes-related neurodegenerative processes (diabetic encephalopathy) at the cellular and molecular levels in a Type 2 diabetic rat model representing the elderly population. Our findings indicate that zinc not only regulates systemic dyslipidemia but also restores redox homeostasis by suppressing oxidative stress (MDA/GSH balance) in hippocampal tissue. Parallel to the reduction in oxidative load, the increased expression of the cellular stress sensor SIRT-1 and the neurotrophic factor brain-derived neurotrophic factor (BDNF) emerges as the primary mechanism protecting neurons from apoptosis (Bax/Bcl-2). Histopathological findings, consistent with biochemical and genetic data, structurally confirm that zinc restricts cellular damage in brain tissue and promotes neuroplasticity (GAP43 and NGF). Therefore, ZnSO_4_ supplementation, which maintains cellular energy balance and preserves synaptic integrity, has multifaceted therapeutic potential against diabetic neurodegeneration. Further studies are required to fully elucidate how these structural and molecular improvements translate into cognitive outcomes and validate these findings with behavioral and cognitive memory tests. Ultimately, the practical and translational goal of this research is to establish ZnSO_4_ supplementation as a safe, accessible, and cost-effective neuroprotective intervention against diabetic encephalopathy, promoting its integration into standard clinical diabetes management to prevent cognitive decline.

## Figures and Tables

**Table 1:** Effects of ZnSO_4_ Treatment on Serum Lipid Profile in an Aged T2DM Rat Model.

Parameters	CON	CON+ZnSO_4_	DM	DM+ZnSO_4_	*p*
Total Cholesterol (mg/dL)	95 ± 8	90 ± 7	185 ± 18 ^a,b^	120 ± 12 ^c^	<0.0001
Triglycerides (mg/dL)	110 ± 10	105 ± 9	260 ± 25 ^a,b^	160 ± 15 ^c^	<0.0001
HDL (mg/dL)	35 ± 4	38 ± 5	12 ± 2 ^a,b^	25 ± 3	0.0001
LDL (mg/dL)	30 ± 4	28 ± 3	95 ± 8 ^a,b^	50 ± 6 ^b,c^	<0.0001

^a^ vs. CON, ^b^ vs. CON+ZnSO_4_, ^c^ vs. DM indicates statistical significance at the *p* < 0.05 level. Data are expressed as Mean ± Standard Error of the Mean (SEM) (*n* = 8). DM, Diabetes Mellitus; ZnSO_4_, Zinc Sulfate; HDL, High-Density Lipoprotein; LDL, Low-Density Lipoprotein.

**Table 2:** Primers used for qPCR.

Gene	Forward Primer Sequence (5′→3′)	Reverse Primer Sequence (5′→3′)
SIRT1	CATAGGTTAGGTGGCGAGTATG	GTTGGTGGCAACTCTGATAAATG
GLUT 3	CATGGGGACAGCGAAGGTGA	ACCGCTCTTCCAACGTGTAG
BDNF	GATCCACTGAGCAAAGCCGA	TCACCTGGTGGAACATTGTGG
Bax	GTTGCCCTCTTCTACTTTG	AGCCACCCTGGTCTTG
BCL-2	CGGGAGAACAGGGTATGA	CAGGCTGGAAGGAGAAGAT
GAPDH	AACTCCCTCAAGATTGTCAGCAA	GGCATGGACTGTGGTCATGA

## Data Availability

The data presented in this study are available on request from the corresponding author. The data are not publicly available due to institutional data-management restrictions and ongoing related research.
